# Supplementary material for: Free Energy Analyses of Cell-Penetrating Peptides Using the Weighted Ensemble Method
Source: Membranes (Basel). 2021 Dec 9;11(12):974. doi: 10.3390/membranes11120974 (PMC8706838; doi:10.3390/membranes11120974)
Supplement: Supplementary file 1 [file membranes-11-00974-s001.zip › membranes-1465938-supplementary.pdf]

Supplementary Information

Free energy analyses of cell-penetrating peptides using the weighted ensemble method

Seungho Choe[1,2]

[1] *Department of Energy Science & Engineering,*

Daegu Gyeongbuk Institute of Science & Technolgy (DGIST), Daegu 42988, South Korea

[2] *Energy Science & Engineering Research Center,*

Daegu Gyeongbuk Institute of Science & Technolgy (DGIST), Daegu 42988, South Korea

I. SYSTEM SETUP

System I and System II were equilibrated for at least 1 μs, while System III and IV were equilibrated for 100 ns. As for System I, we use a pre-equilibrated structure in the previous study [**?**]. Fig. S1 shows snapshots of both the initial and the final structure after finishing equilibration. Each row corresponds to each system (e.g., System I, II, III, and IV). The first and second columns present the initial setup of each system (both a side view and a top view, respectively). Four Arg_9 (or Tat) were initially placed in the upper water box and stayed in the upper water box during equilibration. The third and fourth columns show the final structure after equilibration (both a side view and a top view). A 100ns-long equilibration may be short to fully equilibrate the system (System III and IV); however, a visual inspection shows that four Arg_9 were contacted with the lipid molecules. We also plot radial distribution functions to check the hydration profiles of each system. Fig. S2 presents radial distribution functions g(r) of (a) phosphorus vs. water (b) phosphorus vs. oxygen in water (c) easter oxygen vs. oxygen in water. The radial distribution function of each system is quantitatively very similar to each other, and the structures in System III and IV can be used as initial structures for the WE simulations.

II. MEMBRANE CURVATURE

Fig. S3 shows membrane curvature from System I & II simulations, respectively (left: a side view, right: top view). It indicates significant deformation when Arg_9 or Tat penetrates the DOPC/DOPG(4:1) membrane. Each layer (the upper and the lower) consists of only phosphorus atoms in the lipid molecules in this figure. The colored dots on the upper layer depict C_α atoms of each Arg_9 or Tat.

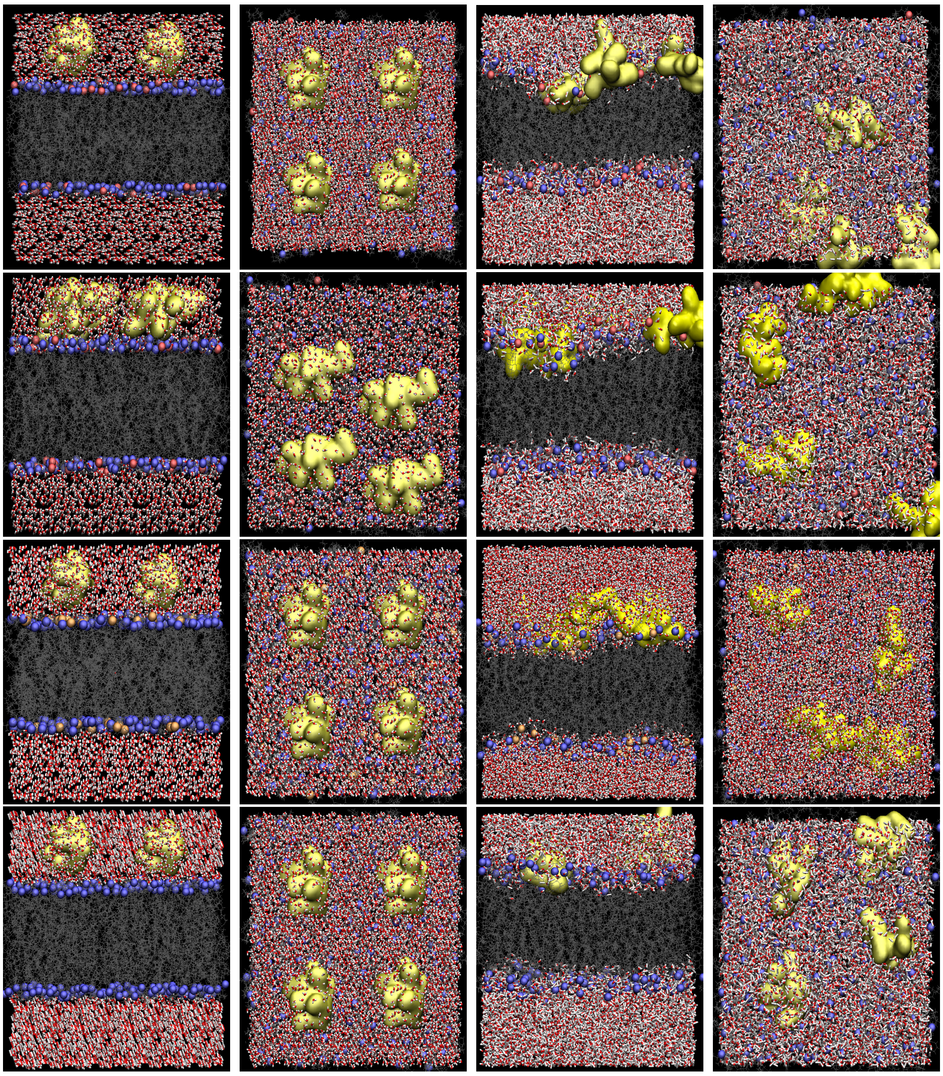

Figure S1: The initial setup and a snapshot after equilibration (first low: System I; second low: System II; third low: System III; fourth low: System IV). The yellow is Arg_9 (System I, III, IV) or Tat (System II). The blue dots depict phosphorus atoms of DOPC lipids, while the red (orange) dots are those atoms of DOPG(DOPE) lipids. The gray line shows lipid molecules, and water molecules are shown as licorice

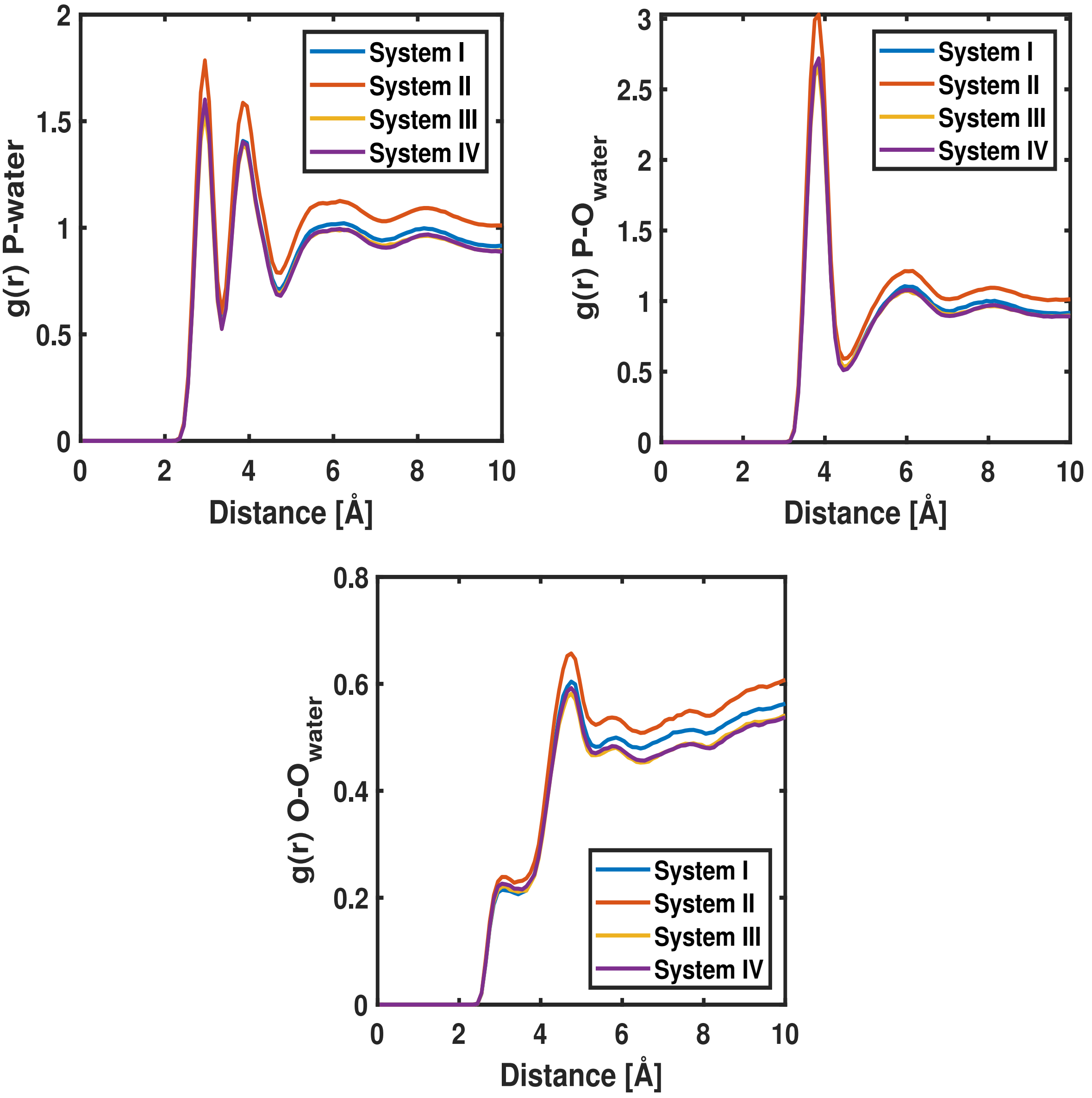

Figure S2: Radial distribution function g(r) of (a) phosphorus atoms in lipids vs. water (b) phosphorus atoms vs. oxygen atoms in water molecules (c) ester oxygen in lipids vs. oxygen atoms in water.

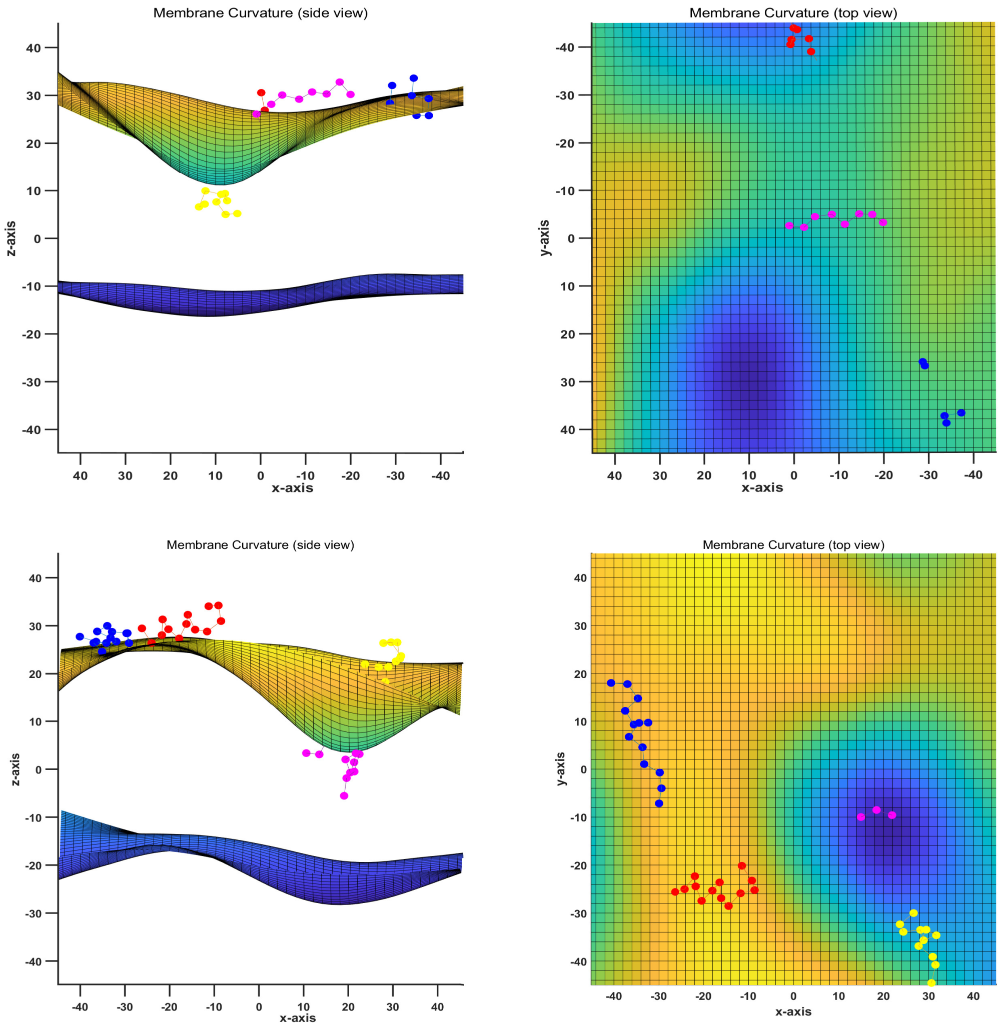

Figure S3: Membrane curvature (a) System I (4 Arg_9 with DOPC/DOPG(4:1) lipids) (b) System II (4 Tat with DOPC/DOPG(4:1) lipids)
